# Recent Advances on Bioinspired Computation

**DOI:** 10.1155/2014/934890

**Published:** 2014-05-06

**Authors:** Zhihua Cui, Rajan Alex, Rajendra Akerkar, Xin-She Yang

**Affiliations:** ^1^Complex System and Computational Intelligence Laboratory, Taiyuan University of Science and Technology, Taiyuan, Shanxi 030024, China; ^2^State Key Laboratory for Novel Software Technology, Nanjing University, Nanjing 210093, China; ^3^West Texas A&M University, Canyon, TX 79016, USA; ^4^Western Norway Research Institute, 6851 Sogndal, Norway; ^5^School of Science and Technology, Middlesex University, London NW4 4BT, UK

Bioinspired computation (BIC) has become an emerging and popular part of modern computer science, artificial intelligence, computational intelligence, and evolutionary intelligence. BIC is usually based on biological systems that can be often self-organizing, adaptive, and tolerant of random defects. Most BIC algorithms are mostly swarm intelligence (SI) based, and they mimic the successful characteristics of biological and swarm systems. However, bioinspired computation can be very broad, including swarm intelligence [1–7], membrane [8] and memetic computing, DNA and molecular computing, neural computing, and others. Their applications are also very diverse, including computational intelligence, computational neuroscience, bioinformatics, natural language processing, machine learning, software engineering, scheduling and timetabling, data mining, algorithm theory, and many other areas [9–11].

Recent advances in this area are relatively extensive, and a special issue can serve as a good summary to provide a snapshot of the recent advances. The responses to our call for papers are overwhelming, and we received nearly two hundred submissions, and after rigorous peer review, only a small fraction of the submissions were accepted for this special issue. This special issue starts with the heterogeneous differential evolution by J. Wang et al., followed by the correction of faulty sensors in phased array radars by J. U. Khan et al. A new variant of harmony search has been proposed to solve binary knapsack problems; then a theoretical model for location of terror response facilities was studied by L. P. Meng et al. In addition, a novel variant of bat algorithm based on naïve Bayes methods has been presented to carry out feature selection, followed by a hybrid model based on genetic algorithms and support vector machine for fruit juice classifications by C. Fernandez-Lozano et al., while a modified dynamic neural-fuzzy approach to model customer satisfaction was carried out by C. K. Kwong et al.

On the other hand, an improved firefly algorithm for nonlinear and convex optimization with applications to CAD/CAM has been proposed by A. Gálvez and A. Iglesias, and an effective hybrid by combining the firefly algorithm and harmony search was found to be efficient for global numerical optimization by L. H. Guo et al. Furthermore, noise-assisted multipath traffic distribution networks were studied by N. Asvarujanon et al. Other studies have focussed on the various improvements and diverse applications with detailed case studies. Interested readers are encouraged to read more about this special issue and the open access mode means that you can download papers and study them in great detail when appropriate.

Analyzing the current developments, we can expect to look forward to the future. From the bioinspired computation point of view, we think the following eight areas need more research.

- Complex, real-world applications: more applications should focus on complex, real-world applications in various engineering and industrial designs. Such applications can be highly nonlinear and multimodal, under stringent constraints.
- Computationally expensive methods: most applications nowadays are computationally expensive because the evaluations of designs, objectives, and computational tasks are becoming computationally extensive. Many design options have to be evaluated by finite element methods, large-scale finite volume methods, and boundary and extended element methods. For example, applications in aerospace and electromagnetic engineering can take hours up to weeks to evaluate computationally. Therefore, further developments in this area to speed up the computational methods are very important for practical applications.
- Data-intensive applications: as the data volumes are expanding dramatically, due to drive in information technology and social media, data-intensive applications become more important. Data mining techniques become more relevant, and bioinspired methods such as PSO, cuckoo search, and firefly algorithm become increasingly popular in such applications [1, 9, 12].
- Network and systems: current research activities have also focused on the modeling and simulation of complex networks and systems such as computer networks, electricity grids, energy networks, and biological systems. Other networks such as social networks have also received some attention. It can be expected that more research will be done in this area.
- Biological applications: bioinspired computation has been applied to biological applications such as protein folding and biological systems [12]. Bioinspired computation has also found applications in bioinformatics and genetic engineering.
- Combinatorial problems: most combinatorial optimization problems such as the traveling salesman problem are NP-hard and thus are very challenging to solve. Bioinspired algorithms such as ant colony optimization and cuckoo search can be very powerful alternatives for dealing with such challenging problems.
- Large-scale problems: the current applications tend to have problem sizes with a few, up to a few dozen, design variables, while real-world applications tend to have thousands of design variables. Therefore, more applications are becoming increasingly large-scale, and it is not yet clear if the methods that work for small-scale problems can still work for large-scale problems. For example, protein folding problems can be very large-scale, and at the moment, nature-inspired methods such as simulated annealing and firefly algorithm have been found to be useful and efficient.
- Data mining and image processing: data mining tends to have data-intensive computation, while image processing can also be very computational extensive as well. Important applications such as feature selection and classifications need efficient methods to solve, especially those large-scale problems.

As we have seen, bioinspired computation is a very active area, and many challenges still remain. It can be expected that more exciting research activities will be seen in the near future.
